# Progress in mosquito net coverage in Papua New Guinea

**DOI:** 10.1186/1475-2875-13-242

**Published:** 2014-06-24

**Authors:** Manuel W Hetzel, Adnan AK Choudhury, Justin Pulford, Yangta Ura, Maxine Whittaker, Peter M Siba, Ivo Mueller

**Affiliations:** 1 Papua New Guinea Institute of Medical Research, PO Box 60, EHP 441 Goroka, Papua New Guinea; 2 Swiss Tropical and Public Health Institute, Socinstrasse 57, PO Box, 4002 Basel, Switzerland; 3 University of Basel, Petersplatz 1, 4003 Basel, Switzerland; 4 The University of Queensland, School of Population Health, 4006 Herston, QLD, Australia; 5 The Walter and Eliza Hall Institute of Medical Research, 1G Royal Parade, 3052 Parkville, VIC, Australia; 6 Barcelona Centre for International Health Research (CRESIB, Hospital Clínic-Universitat de Barcelona), Rosselló 132, 08036 Barcelona, Spain

## Abstract

**Background:**

Since 2004, the Global Fund-supported National Malaria Control Programme of Papua New Guinea (PNG) has been implementing country-wide free long-lasting insecticidal net (LLIN) distribution campaigns. In 2009, after the first distribution, only 32.5% of the population used a LLIN, mainly due to an insufficient number of nets available. This study investigated changes in mosquito net ownership and use following the continued free distribution of LLINs across PNG.

**Methods:**

Five villages from each province and 30 households from each village were randomly sampled in a country-wide household survey in 2010/11. A structured questionnaire administered to household heads recorded information on mosquito net ownership and use alongside household characteristics. Revised ownership and access indicators were applied in the analysis to reveal coverage gaps.

**Results:**

The survey covered 1,996 households in 77 villages. Ownership of at least one LLIN was reported by 81.8% of households, compared to 64.6% in 2009 (*P* = 0.002). Sufficient LLINs to cover all household members (one net per two people) were found in 41.3% of the households (21.4% in 2009, *P* < 0.001). Of all household members, 61.4% had access to a LLIN within their household (44.3% in 2009 *P* = 0.002), and 48.3% slept under a LLIN (32.5% in 2009, *P* = 0.001). LLIN use in children under five years amounted to 58.2%, compared to 39.5% in 2009 (*P* < 0.001). Significant regional differences in coverage and changes over time were observed. A recent LLIN distribution was a key determinant of LLIN ownership (adj. OR = 3.46) while families in high quality houses would frequently not own a LLIN (adj. OR = 0.09). Residents were more likely to use LLINs than household guests (OR = 2.04).

**Conclusions:**

Repeated LLIN distribution has led to significant increases in mosquito net ownership and use with few regional exceptions. Additional nets are required in areas where access is low, while major efforts are required to encourage the use of existing nets in region where access is high but use remains low. Complementary vector control approaches should also be considered in such settings.

## Background

Papua New Guinea (PNG) is a malaria-endemic country in the South-West Pacific with a population of approximately seven million (2011). Financial support from the Global Fund to Fight AIDS, Tuberculosis and Malaria has enabled the National Malaria Control Programme (NMCP) to roll out long lasting insecticidal nets (LLIN) country-wide since 2004 alongside complementary malaria control interventions [1,2]. The first round of free LLIN distribution between 2004 and 2009 resulted in a significant increase in household ownership of LLINs across the country. Nevertheless, average LLIN use the night prior to the survey remained at only 32.5% in 2009 and hence well below the 80% programme target [1]. Subsequent studies identified a lack of sufficient nets, heterogeneous distribution as well as indifference towards malaria as the main reasons behind the low usage levels [1,3]. Ownership on the other hand was largely a function of the accessibility of villages with remote locations showing significantly lower household ownership of LLINs [1]. Eighty-seven percent of the PNG population still live in rural areas, often in villages that lack road access and are reachable only by air, foot, or boat [4]. As a result of uneven development since the time of colonization [5], many people living in remote rural areas are deprived of basic services and infrastructure, such as electricity or piped water, despite a wide network of government stations.

Gaps in mosquito net coverage found after the first free distribution of LLINs (2004–2009) were to be addressed during subsequent country-wide distribution campaigns (2010–2014), funded by a Global Fund Round 8 grant and under the operational responsibility of the non-governmental organization, Rotarians Against Malaria (RAM). The distribution ratio remained at one net per 2.5 household members, which in operational practice required a population census to be carried out in each village prior to sending the campaign team with nets.

Six years after the first country-wide distribution campaign and two years into the second Global Fund grant (Round 8), a country-wide household survey was conducted with the aim of tracking progress in malaria control intervention coverage and identifying gaps in net ownership and use across different population groups and socio-economic strata. For this purpose, a set of old and new indicators were compared with the baseline survey conducted in 2008/09, the methodology and results of which are described in detail elsewhere [1].

## Methods

### Study design

A country-wide household survey based on the methodology of the Malaria Indicator Survey [6] and following the design of the 2008/09 malaria survey [1] was conducted in 17 out of 20 provinces of PNG between November 2010 and August 2011. The survey was discontinued prior to covering all provinces following the disappearance of an entire survey team with five PNG Institute of Medical Research staff members in West New Britain Province in August 2011 [7].

Sampling of survey households was based on a province-stratified multi-stage sampling approach. A random sample of five villages per province was drawn from a geo-referenced census database [8]. Two back-up villages were sampled in case one of those initially sampled could not be surveyed. Within each village, 30 households were randomly selected from a household list established *ad hoc* by the survey team leader and village representatives. In villages with less than 30 households, all households were included. A household was excluded if after three separate attempts there was no adult household resident available to provide consent and information.

### Data collection

Three trained field teams, each led by a scientific officer (BA/BSc graduates), worked simultaneously at different sites across PNG administering a structured questionnaire to the adult heads of sampled households. Following the design of the Malaria Indicator Survey Household Questionnaire [6], this instrument was used to record household characteristics, demographic information of household members (residents and guests) as well as household ownership and individual use of mosquito nets. Interviewees were asked about exposure to behaviour change messages about malaria in the past three months. Village locations and elevation above sea level were recorded with hand-held GPS devices (Garmin etrex, Garmin Ltd., Olathe, Kansas, USA).

### Data analysis

Random selection of villages was performed using Stata 8.1 software (StataCorp LP, College Station, USA). All data were double-entered into a Visual Foxpro 9.0 (Microsoft) or DMSys (SigmaSoft International) database at PNG IMR Goroka and analysed with Stata 12.1.

Aggregated national and regional level weighted proportions with logit transformed 95% confidence limits were calculated using the survey design command set in Stata. Overall sampling weights were calculated as the inverse of an observation’s probability of selection. To account for the staged sampling design, the overall probability of selection was calculated as a product of the selection probabilities at each sampling stage, i.e. the probability of a village being selected within a district and the probability of a household being selected within a village. Since all individuals of the sampled household were eligible, individual level weights equalled the weights of the households to which an individual belonged. Household and individual level coverage indicators included those proposed for the evaluation of malaria control programmes [9,10]. Ownership of more than one LLIN per household and LLIN use in the target groups of children under five years and pregnant women were key indicators for the Global Fund grant evaluation [2]. One LLIN per two people was considered to be sufficient, on average, to protect all individuals in the household [11,12]. The proportion of the population with access to a LLIN within their household was calculated by dividing the number of LLIN sleeping spaces (two per LLIN) by the number of people sleeping in the household and then multiplied each household observation by the number of people in the household the previous night [10]. Ownership and use gaps were calculated for selected background characteristics as proposed by Kilian *et al.*[13] as the inverse of “the proportion of households owning a LLIN”, “the proportion of households with sufficient LLINs” (as defined above) and “the proportion of people with access using an LLIN”. The latter indicator was calculated by dividing the number of people using an LLIN by the total population with access (derived from applying the weighted proportion with access to the total population). This approach was required as the access indicator is calculated at a household level and does not allow allocation of access to individuals, as previously discussed by Kilian *et al.*[13].

In order to test for equitable outcomes of the net distribution across socio-economic strata, an asset index was constructed using the principal component analysis (PCA) function of Stata. The PCA included parameters such as house wall type, lighting source, type and number of livestock and household assets owned. Education and occupation were dropped from the final PCA due to the generation of statistical anomalies. A full list of variables used in the asset index and in the construction of a “high quality house” variable is available as supplementary file (see Additional file 1).

Bivariate analyses included chi-square tests to assess dichotomous variables and adjusted Wald tests to compare means between groups. Predictor selection for multivariate logistic regression models was based on a manual backward selection including all variables tested by univariate analysis with those with *P* < 0.2 being retained in the model.

### Ethical considerations

The study protocol was approved by the Institutional Review Board of PNG IMR (IMR IRB No. 0933) and the Medical Research Advisory Committee (MRAC No. 10.12). Permissions to conduct this study were also obtained from relevant provincial and local authorities, village leaders and household heads.

## Results

### Study sample

The survey was conducted in 77 villages located in 17 provinces and included 1,996 households with 12,548 individuals present the night prior to the interviewer’s visit. Details of the study sample are presented in Table 1 alongside population estimates for the four geographical regions. An additional five household interviews were excluded from the analysis due to incomplete data and 1,366 additional household members were reportedly not present in the surveyed households the previous night and hence not considered part of the *de facto* household population.

**Table 1:** Projected population and study sample by geographical region

**Region**	**2011 population projection**^**§**^	**Provinces covered/Total**	**Villages**	**Households**	**Individuals present the previous night**	**Average *de facto *** **household size**
	**N (%)**	**N**	**N (%)**	**N (%)**	**N (%)**	**N**
Southern	1,426,636 (20.3)	6/6	27 (35.1)	784 (39.3)	5,274 (42.0)	6.4
Highlands	2,706,401 (38.6)	4/5*	18 (23.4)	434 (21.7)	2,417 (19.3)	5.5
Momase	1,897,403 (27.0)	4/4	17 (22.1)	396 (19.8)	2,681 (21.4)	6.8
Islands	987,259 (14.1)	3/5**	15 (19.5)	382 (19.1)	2,176 (17.3)	5.8
Total	7,017,699	17/20	77	1,996	12,548	6.1

^§^Based on year 2000 national census, *Excluding Eastern Highlands, **Excluding West New Britain, Bougainville.

The median *de facto* household population was 6 (interquartile range 4, 7). In 7.1% of all households, two individuals or less were present the previous night. The individual sample included 1,782 children below five years of age (14.2% of all individuals with reported age) and 120 pregnant women aged 15–49 years (3.8% of women in this age group). For 26 (0.20%) individuals, no age information was available. Of all individuals present in the household the previous night, 2.2% were not residents but temporary visitors of the surveyed households.

### Mosquito net ownership

Across PNG, 81.8% (95% CI 74.5, 87.3) of households reported owning at least one LLIN, 66.3% (95% CI 57.7, 73.9) more than one LLIN, and 86.8% (95% CI 79.3, 91.8) any type of mosquito net. The proportion of households with at least one LLIN for every two people reached 41.3% (95% CI 34.5, 48.5). Among households owning at least one LLIN in 2011, 50.6% (95% CI 44.8, 56.4) had a sufficient number of these nets. The presence and type of 5,221/6,066 reported nets were confirmed by visual inspection. The mean number of *de facto* household members was found to be significantly higher in households without sufficient LLINs than in households with sufficient LLINs (6.9 *vs.* 4.9, *P* < 0.001), as was the number of non-residents (visitors) in the household the previous night (0.17 *vs.* 0.04, *P* <0.001). Key indicators of LLIN ownership by background characteristics are presented in Table 2.

**Table 2:** Mosquito net ownership in 2011

**Background characteristic**	**Ownership of LLIN,**	**Ownership of >1 LLIN,**	**One LLIN per two people,**	**Number of households**
	**% (95% CI)**	**% (95% CI)**	**% (95% CI)**	
**Region**				
Southern	94.3 (91.6, 96.2)	85.8 (82.5, 88.6)	61.6 (55.7, 67.2)	784
Highlands	76.1 (62.5, 85.8)	57.1 (41.2, 71.8)	36.0 (23.6, 50.7)	434
Momase	75.4 (62.9, 84.8)	59.7 (49.2, 69.3)	25.9 (18.4, 35.1)	396
Islands	98.3 (95.0, 99.4)	84.5 (79.0, 88.8)	62.2 (57.6, 66.6)	382
* P*-value	*0.002*	*0.001*	*<0.001*	
**Road access**				
No	90.9 (83.1, 95.3)	75.2 (67.6, 81.5)	46.4 (38.2, 54.8)	783
Yes	78.1 (68.7, 85.3)	62.7 (51.2, 72.9)	39.3 (30.2, 49.3)	1,213
* P*-value	*0.018*	0*.*057	0.304	
**Altitude (m)**				
0-1299	88.0 (82.9, 91.7)	75.4 (70.8, 75.4)	47.3 (42.3, 52.3)	1,538
1300-1699	65.4 (42.0, 83.1)	47.8 (27.8, 68.5)	28.3 (15.2, 46.5)	122
1700+	78.9 (61.8, 89.6)	59.9 (39.2, 77.5)	37.6 (21.8, 56.6)	336
* P*-value	0.055	0.056	0.223	
**LLIN distribution**				
>2 years ago or never	67.6 (55.0, 78.2)	48.4 (34.9, 62.2)	25.1 (16.0, 36.9)	433
Last 2 years	88.8 (84.3, 92.2)	75.1 (70.1, 79.6)	49.5 (44.3, 54.7)	1,544
* P*-value	*<0.001*	*<0.001*	*<0.001*	
**High quality house**				
No	82.3 (74.9, 87.9)	66.7 (57.8, 74.5)	42.0 (35.0, 49.3)	1873
Yes	56.0 (37.6, 72.8)	46.3 (31.4, 61.8)	11.5 (5.6, 22.3)	123
* P*-value	*0.002*	*0.020*	*<0.001*	
**Wealth quintile**				
Lowest	76.0 (63.2, 85.4)	61.6 (45.2, 75.7)	37.9 (25.1, 52.6)	387
Second	80.1 (70.7, 87.0)	59.6 (50.8, 67.9)	35.4 (28.4, 43.1)	386
Third	86.5 (80.4, 91.0)	70.9 (60.8, 79.3)	49.7 (39.5, 59.9)	387
Fourth	86.4 (79.0, 91.5)	76.0 (69.0, 81.9)	48.9 (41.0, 56.9)	386
Highest	78.1 (65.8, 86.9)	63.9 (53.1, 73.5)	31.7 (22.6, 42.5)	386
* P*-value	*0.045*	*0.031*	*0.030*	
**Total**	**81.8 (74.5, 87.3)**	**66.3(57.7, 73.9)**	**41.3(34.5, 48.5)**	**1,996**

*P-*values of chi^2^ tests.

**Figure 1:**
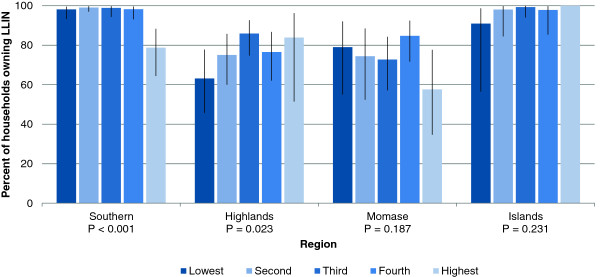
**Household ownership of at least one LLIN by region and wealth quintile.** Error bars represent 95% confidence intervals.

Based on a multivariate logistic regression analysis, households in areas covered by a distribution campaign in the previous two years (i.e. over the course of the Global Fund Round 8 grant) were significantly more likely to own at least one LLIN and also sufficient LLINs than households not covered within that period (Table 3). Families in high quality houses (all of whom belonged to the highest wealth quintile) were substantially less likely than others to own at least one LLIN or sufficient LLINs for all household members (adjusted odds ratio [adj. OR] = 0.09 and 0.10, respectively). Households located at intermediate and high altitudes (≥1300 m) were less likely to own a LLIN or sufficient LLINs, independent of the time of LLIN distribution. The number of household members was negatively correlated with ownership of sufficient nets. Households in the third and fourth wealth quintile were significantly more likely than households in the lowest quintile to own at least one LLIN (adj. OR = 1.86, *P* = 0.031 and adj. OR = 2.00, *P* = 0.014, respectively) but no differences were found in ownership of sufficient LLINs. A region-stratified analysis of LLIN ownership revealed statistically significant differences between wealth quintiles only in Southern and Highlands regions (Figure 1). Some regional differences remained after adjusting for other predictors, notably the lower odds of owning sufficient LLINs in the Islands region, which contrasts with the high proportion of households with sufficient nets (62.2%) found there. After adjusting for all other factors in the multivariate analysis, no significant differences were found between villages with or without road access (Table 3).

**Table 3:** Multivariate analysis of predictors of household LLIN ownership

**Predictor**	**Ownership of LLIN**			**One LLIN per two people**		
	**Adj. OR**	**(95% CI)**	***P*-value**	**Adj. OR**	**(95%CI)**	***P*-value**
**Region**						
Southern	1		*<0.001*	1		*<0.001*
Highlands	1.26	(0.28, 5.60)		1.32	(0.37, 4.70)	
Momase	0.25	(0.11, 0.55)		0.26	(0.12, 0.58)	
Islands	1.68	(0.42, 6.65)		0.59	(0.41, 0.85)	
**Road access**						
No	1					
Yes	0.58	(0.25, 1.34)	0.198			
**Altitude (m)**						
0-1299	1		*<0.001*	1		*<0.001*
1300-1699	0.18	(0.08, 0.38)		0.19	(0.09, 0.42)	
1700+	0.16	(0.04, 0.65)		0.17	(0.04, 0.76)	
**LLIN distribution**						
>2 years ago or never	1			1		
Last 2 years	3.46	(1.93, 6.22)	*<0.001*	2.82	(1.41, 5.63)	*0.004*
**Recent BCC exposure**						
No				1		
Yes				0.69	(0.47, 1.01)	0.054
**Household size**						
Mean number of *de facto* members	1.06	(0.98, 1.14)	0.145	0.68	(0.62, 0.75)	*<0.001*
**High quality house**						
No	1			1		
Yes	0.09	(0.03, 0.27)	*<0.001*	0.10	(0.03, 0.32)	*<0.001*
**Wealth quintile**						
Lowest	1		0.102	1		0.068
Second	1.27	(0.85, 1.88)		1.09	(0.72, 1.67)	
Third	1.48	(0.91, 2.41)		1.86	(1.06, 3.25)	
Fourth	1.42	(0.87, 2.31)		2.00	(1.16, 3.44)	
Highest	0.75	(0.34, 1.63)		1.31	(0.70, 2.46)	

### Mosquito net use

The proportion of household members with access to a LLIN within their household reached 61.4% (95% CI 55.3, 67.4) (Table 4). This resulted in 48.3% (95% CI 41.8, 54.9) of the population sleeping under a LLIN the previous night and 54.8% (95% CI 47.9, 61.5) under a mosquito net of any type. LLIN use in the target group of children below five years of age amounted to 58.2% (95% CI 50.8, 65.3), in the group of pregnant women to 49.9% (95% CI 39.6, 60.1). Of 275 infants below one year of age, 72.2% (95% CI 61.6, 80.7) slept under a LLIN. Indicators of access to LLINs and their use by background characteristics are presented in Table 4.

**Table 4:** Access to and use of LLINs in 2011

**Background characteristic**	**Access to LLIN, % (95%CI)**	**LLIN use, % (95%CI)**	**Population**
**Region**			
Southern	77.8 (73.2, 82.3)	67.2 (56.6, 76.3)	5,274
Highlands	55.1 (43.9, 66.2)	39.6 (29.3, 50.9)	2,417
Momase	50.9 (39.0, 62.8)	48.7 (35.7, 61.9)	2,681
Islands	81.6 (78.5, 84.7)	39.9 (32.8, 47.5)	2,176
* P*-value	*<0.001*	*0.004*	
**Road access**			
No	69.6 (62.1, 77.1)	57.3 (47.2, 66.8)	4,731
Yes	57.9 (50.0, 65.8)	44.6 (36.8, 52.6)	7,817
* P*-value	*0.044*	0.051	
**Altitude (m)**			
0-1299	67.4 (61.8, 72.9)	55.7 (48.1, 63.1)	9968
1300-1699	45.6 (28.5, 62.8)	34.4 (24.7, 45.5)	687
1700+	57.0 (43.3, 70.7)	40.9 (28.0, 55.3)	1893
* P*-value	*0.043*	*0.030*	
**LLIN distribution**			
>2 years ago or never	47.6 (36.7, 58.4)	42.4 (30.6, 55.2)	2,680
Last 2 years	67.8 (62.9, 72.7)	51.1 (44.6, 57.6)	9,868
* P*-value	*0.001*	0.212	
**Recent BCC exposure**			
No	61.2 (54.2, 68.1)	47.9 (40.9, 55.0)	10,087
Yes	62.7 (53.9, 71.5)	50.5 (39.7, 61.1)	2,406
* P*-value	0.783	0.668	
**High quality house**			
No	62.1 (56.0, 68.3)	49.3 (42.7, 55.9)	11,570
Yes	35.3 (23.1, 47.5)	15.4 (8.4, 26.4)	978
* P*-value	*<0.001*	*<0.001*	
**Wealth quintile**			
Lowest	61.1 (50.6, 71.6)	52.3 (39.3, 65.0)	2,136
Second	58.7 (50.9, 66.5)	51.3 (43.3, 59.3)	2,217
Third	65.8 (56.8, 74.9)	54.5 (45.1, 63.5)	2,272
Fourth	66.3 (59.0, 73.6)	47.9 (40.1, 55.8)	2,406
Highest	52.5 (42.3, 62.7)	32.8 (24.3, 42.6)	3,125
* P*-value	0.076	*0.010*	
**Household head schooling**			
No	54.3 (45.2, 63.4)	42.4 (34.3, 51.0)	1,788
Yes	63.1 (57.4, 68.7)	50.2 (43.5, 56.8)	10,207
* P*-value	*0.014*	*0.029*	
**Residence**			
Does not live here	NA	28.5 (19.9, 39.1)	279
Lives here	NA	48.7 (42.2, 55.3)	12,265
* P*-value		*0.001*	
**Age group (years)**			
<5	NA	58.0 (50.8, 65.3)	1,782
5-9	NA	51.3 (42.8, 59.7)	1,883
10-14	NA	49.4 (42.2, 56.6)	1,503
15-19	NA	42.0 (35.1, 49.2)	1,332
20+	NA	45.7 (39.4, 52.1)	6,022
* P*-value		*<0.001*	
**Sex**			
Female	NA	50.1 (43.6, 56.6)	6,243
Male	NA	46.6 (39.9, 53.5)	6,269
* P*-value		*0.004*	
**Total**	**61.4 (55.3, 67.4)**	**48.3 (41.8, 54.9)**	**12,548**

NA = not applicable, as the indicator is calculated at a household level and cannot be allocated to individuals. *P-*values of chi^2^ tests.

While LLIN ownership was highest in the Islands and Southern regions (Table 2), with a resulting high proportion of the population having access to a LLIN within their household (77.8% in Southern, 81.6% in the Islands), it translated to high LLIN use only in Southern region (67.2%) but remained low in the Islands (39.9%). Regional differences were statistically significant (Table 4). Adjusted for potential confounders, people in the Islands and Momase regions were significantly less likely to sleep under a mosquito net than people in Southern region (adj. OR = 0.19, *P* < 0.001 and adj. OR = 0.45, *P* = 0.012, respectively) (Table 5). At altitudes above 1300 meters, LLIN use was substantially less common than in lower-lying locations. LLIN use differed slightly between wealth quintiles with people in the highest quintile having less access (Table 4) and being less likely to use a LLIN than people in the lowest quintile (adj. OR = 0.56, *P* = 0.055). People sleeping in a high quality house were far less likely to have access and to use a LLIN than others (adj. OR = 0.11) and residents of households were twice as likely to use a LLIN as guests (adj. OR = 2.04). Female household members were slightly more likely to use a LLIN (adj. OR = 1.17) and children below five years of age were more likely to use a LLIN than older age groups (Table 5).

**Table 5:** Multivariate analysis of predictors of LLIN use

**Predictor**	**LLIN use**		
	**Adj. OR**	**(95%CI)**	***P*-value**
**Region**			
Southern	1		*<0.001*
Highlands	0.62	(0.23, 1.63)	
Momase	0.45	(0.24, 0.83)	
Islands	0.19	(0.11, 0.32)	
**Altitude (m)**			
0-1299	1		*<0.001*
1300-1699	0.32	(0.18, 0.57)	
1700+	0.37	(0.14, 1.00)	
**LLIN distribution**			
>2 years ago or never	1		
Last 2 years	1.50	(0.93, 2.43)	0.096
**Household size**			
Mean number of *de facto* members	0.90	(0.86, 0.95)	*<0.001*
**High quality house**			
No	1		
Yes	0.11	(0.05, 0.26)	*<0.001*
**Wealth quintile**			
Lowest	1		*0.048*
Second	1.03	(0.96, 1.52)	
Third	1.17	(0.82, 1.69)	
Fourth	1.00	(0.59, 1.69)	
Highest	0.56	(0.31, 1.01)	
**Household head schooling**			
No	1		
Yes	1.27	(0.94, 1.71)	0.119
**Residence**			
Does not live here	1		
Lives here	2.04	(1.17, 3.55)	*0.013*
**Age group (years)**			
<5	1		*<0.001*
5-9	0.73	(0.59, 0.91)	
10-14	0.68	(0.52, 0.90)	
15-19	0.49	(0.36, 0.67)	
20+	0.57	(0.48, 0.67)	
**Sex**			
Male	1		
Female	1.17	(1.05, 1.30)	*0.004*

### Trends and gaps in LLIN ownership and use

**Figure 2:**
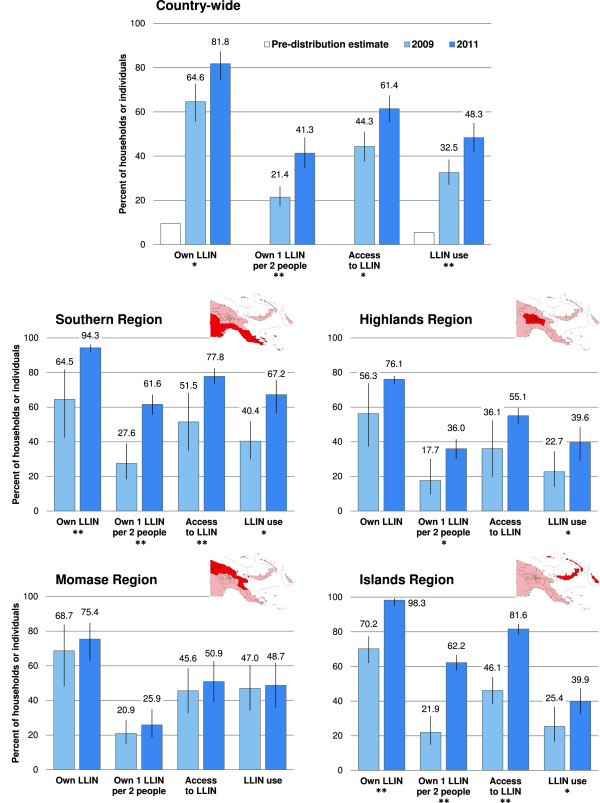
**National (top) and regional changes in LLIN ownership and use between surveys in 2009 and 2011.** Region highlighted in red on maps. Error bars represent 95% confidence intervals. Pre-distribution estimate and 2009 data derived from Hetzel *et al.*[1]. Statistical significance of differences in indicators between years: *P < 0.05, **P < 0.001.

Between the surveys in 2008/09 and 2010/11, statistically significant increases were found in household ownership of at least one LLIN and of sufficient LLINs at country-level (*P* = 0.02 and *P* < 0.001, respectively) (Figure 2). The increase in LLIN use in the general population and in children under the age of five years was also significant (*P* = 0.001 and *P* < 0.001, respectively), but not so in the target group of pregnant women (*P* = 0.255) (data for children and pregnant women not shown in Figure 2).Ownership and use indicators changed to different degrees in the four geographical regions. In Southern and Islands regions, ownership, access and use increased significantly (Figure 2). Major and significant increases in the proportion of households with at least one LLIN for every two people were found in all regions except Momase. The same was observed for LLIN use, albeit reaching only borderline statistical significance in the Highlands. In general, no significant changes in indicators were found in Momase region, but 2011 values for several indicators remained comparable to the other regions. In the Islands region, near-universal spatial coverage (98.3% ownership of at least one LLIN) and very high access (81.6%) did not translate into high use (39.9%) (Figure 2).

Across PNG, the remaining gap in spatial coverage in 2011 amounted to 18.2% (95% CI 12.7, 25.5) of households having no LLIN at all. This proportion was generally lower in villages covered with a recent net distribution campaign (11.2%, 95% CI 7.9, 15.8, *P* < 0.001). A village-level analysis reveals important differences in ownership between survey locations. Of the 77 surveyed villages, 29.9% had reached 100% household ownership and 74.0% of villages 80% or more. Of six (7.8%) survey locations with <50% ownership, four were urban areas (in Port Moresby and Lae). The Global Fund year 2 (2010) target of 61% ownership of two or more LLIN per household was reached in 68.8% of surveyed villages and in 91.5% of 59 villages covered with a recent (Round 8) distribution campaign. The final (year 2014) programme target of 90% ownership of two or more LLINs was reached in 23.4% of surveyed villages and in 28.8% of recently covered villages.

Among households with at least one LLIN, 49.4% (95% CI 43.6, 55.2) did not own a sufficient number of these nets to cover all household members (one LLIN per two people). This proportion amounted to 44.2% (95% CI 39.0, 49.6) in areas recently covered with a distribution campaign. Insufficient numbers of nets resulted in 38.6% of the people not having access to a LLIN within their household. Of those people having access to a LLIN in their household, 78.7% (95% CI 69.9, 78.3) had made use of it the previous night. The proportion of people with access not using a LLIN amounted to 51.1% in the Islands, 28.1% in the Highlands, 13.6% in Southern and 4.3% in Momase region, but did not differ notably between households with (19.4%) and without (21.7%) self-reported recent exposure to malaria BCC messages. In 51.9% of surveyed villages, the Global Fund year 2 target of 60% LLIN use by children below five years of age was reached. A 60% use by the general population was reached in only 35.1% of the villages.

### Reasons for non-use

Of 5,361 LLINs registered in surveyed households, 2,143 had not been used the previous night. The most frequently cited reasons why a particular LLIN was not used included: the net being spared, e.g. for later use (37.0%), the owner of the net being absent (9.9%), the perceived absence of mosquitoes (9.0%), heat (8.9%), and general indifference or opposition towards the use of mosquito nets (6.5%). Only rarely was re-purposing the given reason for not using a particular LLIN at night (0.1%). In the Islands region, where the discrepancy between access and use was largest, the primary reasons for a LLIN remaining un-used included: heat (19.8%), the net being spared (19.2%), the perceived absence of mosquitoes (16.3%) and indifference or opposition towards net use (13.1%). Heat was also commonly mentioned as a reason for non-use in Southern region (12.9%) (Table 6).

**Table 6:** Most important reported reasons for non-use of registered LLINs by region

**Reported reason**	**Percent of un-used LLINs**					***P*-value**
	**Southern**	**Highlands**	**Momase**	**Islands**	**Overall**	
Net spared/reserved	55.8	28.0	45.4	19.2	37.0	*<0.001*
User did not sleep here	7.2	13.5	11.0	6.2	9.9	0.165
No mosquitoes	9.4	5.8	6.7	16.3	9.0	0.187
Too hot	12.9	1.3	5.8	19.8	8.9	*0.002*
Indifferent/opposed to net use	3.0	7.0	3.3	13.1	6.5	*0.006*
Net expired/damaged	3.1	4.4	9.3	0.3	3.9	*0.022*
House factors/space	0.5	3.2	0.0	3.4	2.0	0.071
Net dirty/being washed	0.0	3.6	0.6	2.3	1.9	0.186
Other vector control method preferred	1.3	2.0	0.6	0.9	1.4	0.690
Perceived absence of malaria	0.0	2.9	0.0	0.2	1.1	0.076
Afraid of smoke/fire damaging the net	0.0	2.1	0.6	1.1	1.1	0.141

Reasons reported for less than 1% of nets are not displayed.

## Discussion

This survey represents the second country-wide assessment of the coverage with mosquito nets since the start of Global Fund-supported LLIN distributions in 2004. The first survey in 2008/09 found that ownership and use fell short of the targets defined in the Global Fund Round 3 grant performance framework despite substantial improvements in coverage compared to the pre-distribution situation [1]. Results in this manuscript provide the evidence of a significant increase in country-level LLIN ownership and use indicators since 2009, resulting in a high proportion of households (81.8%) owning at least one LLIN with a peak in the Islands region (98.3%). From a programme performance point-of-view, the year 2 target of 61% household ownership of more than one LLIN was met at country-level (66.3%) and within 95% confidence limits in all regions. Current ownership is beyond what has been reported as average LLIN ownership for sub-Saharan Africa (56% in 2012) [14] and comparable with the levels achieved in African countries with a long history of mosquito net use (e.g. Tanzania, 63.4% in 2010, preliminary 91.5% in 2012) [15]. However, only 41.3% of households in PNG owned a sufficient number of LLINs (defined as one LLIN per two people) ranging from 25.9% in Momase to 62.2% in the Islands region. This proportion was slightly higher (50.6%) when considering only LLIN-owning households.

Households in villages recently covered with a LLIN distribution campaign were far more likely than others to own at least one LLIN (adj. OR = 3.46) or sufficient LLINs (adj. OR = 2.24), providing clear evidence of the impact of the free distribution on coverage. Whether this was a result of repeated or single campaign coverage could not be established in the frame of this study, mainly due to ambiguous reporting of previous distribution campaigns by village leaders. It is however well known that the number of effective LLINs in households declines over time as a result of loss, net attrition, damage, and insecticide decay, amongst other factors, at rates dependent on the epidemiological setting and contextual factors, such as net use and wash patterns [16-20]. It has been suggested that an attrition of 50% of nets can be expected after three years [21]. In certain contexts, re-purposing of nets may also play an important role [22] as well as the population growth since the last campaign.

Even in areas recently covered with a campaign and with 88.8% household LLIN ownership, only 45.8% of households owned sufficient LLINs to cover everybody at a ratio of one net per two people. The net-per-person ratio is supported by an analysis of 35 datasets from sub-Saharan Africa that found a median of two people sleeping under an insecticide-treated net [12]. In PNG, mosquito nets were distributed on a needs basis taking into consideration age- and sex-composition of households for predicting sleeping arrangements within each household. This approach was considered suitable for reducing both over- and under-supply of LLINs (Tim Freeman, RAM, personal communication). In the previous Round 3 distribution (2004–2009), LLINs had been distributed at a rate of one net per 2.5 household members, which could be expected to result in under-supply unless a sufficient number of usable pre-existing nets were present in households. While it has been suggested that pre-existing nets may be taken into account during repeated distributions, this should be done only where pre-campaign ownership of nets less than two years of age exceeds 40% [23]. In practice, this would refer to locations in which a distribution campaign in the previous two years had achieved high coverage and would require a pre-distribution assessment of the number of LLINs present in each household. It has been shown in the past that mass campaigns tend to systematically supply insufficient nets to reach universal coverage [24]. It appears that in PNG, both the previous distribution ratio, as well as the current needs-based strategy provided insufficient LLINs particularly to larger households (Tables 2 and 3). A more practical solution to increasing ownership of sufficient nets would potentially be an adjustment of the net distribution ratio to at least one LLIN for every two household members – even if this resulted in over-supply in some households. A better understanding of the “life history” of mosquito nets as well as the number of users per net in the PNG context would help in the interpretation of these findings and in the planning for future distributions.

In 2009, difficult village accessibility was the main obstacle to household LLIN ownership [1], while in 2011 accessibility of villages did not appear as a significant predictor of LLIN ownership any longer. While these results suggest a better penetration of the recent campaign to remote areas, it should be noted that, contrary to the 2008/09 survey, the random sample of this recent survey did not include any village only accessible by airplane. Interestingly, the lowest ownership levels were found in urban areas, and families in “high quality houses” were significantly less likely to own at least one LLIN or sufficient LLINs (both adj. OR = 0.09). Lower mosquito net coverage in urban centres is regularly observed in household surveys, e.g. [25,26]. In PNG, this may be a result of difficulties in distributing to households in urban areas, where more people had formal employment and were not reachable during the day. In urban locations, RAM distributed net vouchers which could be exchanged against LLINs in a central location in the neighbourhood (Tim Freeman, RAM, personal communication). However, this study found no clear correlation between source of household income (wage job *vs*. others) and LLIN ownership after adjusting for “high quality house”. The observed situation might hence also be related to people in high quality houses feeling less of a need to collect or to retain mosquito nets.

In the 2008/09 survey, ownership was found to be the main determinant of use with 99.5% of non-users not having access to a spare net in their household [1]. Provision of additional LLINs was, therefore, required and free distribution campaigns complemented with free provision of LLINs to pregnant women during antenatal care visits were identified as preferred strategies for the PNG setting. Behaviour change campaigns emphasizing the health benefit of regular use of LLINs were expected to contribute to an increase in LLIN use. This survey found that the use of LLINs had increased significantly over the previous two years (+48.6%), even more than access to a LLIN had increased over the same period (+38.6%). Access levels reached in villages recently covered by a campaign (67.8%) were at the upper end of those reported from recent household surveys in sub-Saharan Africa (ranging from 11 to 74%) [14]. Nevertheless, a significant gap remained in certain settings between access to a LLIN and actual use. Overall, 78.7% of people with access to a LLIN made use of this opportunity, which was less than the 88% reported from African countries [14]. Particularly in the Islands region, where the population prevalence of malaria remains high [27], over 50% of people do not make use of available LLINs. Indifference has been identified as an important impediment to using an available mosquito net in PNG [3]. This survey confirmed the importance of personal choice (e.g. sparing the net for later use), risk perception and environmental factors (e.g. heat). In the Islands, perceived absence of risk and personal indifference/opposition towards nets alongside heat-related concerns may to a large extent explain the low usage. Behaviour change campaigns (BCC) could not be shown in this survey to have an impact on current net use. This may be related to only moderate BCC coverage: 14.4% of households across PNG reported exposure to behaviour change messages on malaria from any source in the last three months; in the Islands region this amounted to 29.9% (Hetzel *et al.*, PNG IMR, unpublished data). The role of household visitors, found to frequently not use a LLIN, has so far not come up prominently in the literature. In surveys on mosquito net coverage, visitors present the previous night are generally considered part of the *de facto* household population and included in the calculation of net use. However, they would not be considered in the calculation of required nets during a distribution campaign. In 2008/09, a prominently reported reason for not using a particular net was the absence of the net owner [1], indicating that people do not necessarily carry their mosquito nets when travelling. This, in combination with a lack of spare nets in households, leaves household visitors (2.2% of the surveyed population) more exposed than residents.

Most areas categorized as recently covered with a free distribution campaign are scheduled to be covered once more over the course of the Round 8 Global Fund grant. Additional nets in already covered areas are important to meet the final Global Fund targets, as only 28.8% of recently covered villages had reached 90% household ownership of two or more LLINs. Particularly larger households appear to require more LLINs as evidenced by the negative correlation of household size and ownership of sufficient nets (adj. OR = 0.68). Ownership of nets across the country is expected to increase further with another distribution round, particularly considering that LLINs are expected to remain usable for several years. A recent study carried out in PNG found that LLINs used for five years and more had retained high bio-efficacy against local anophelines [20]. However, the physical condition of nets was found to degrade significantly after five years in use and drying of nets in the sun was associated with a significant reduction in insecticide concentration, supporting the recommendation to replace LLINs after not more than five years. In a qualitative study even relatively new nets were sometimes found to be damaged beyond repair and consequently considered unusable by their owners [3]. Quantitative data about the life span of nets under different conditions of use combined with in-depth data about the relationship of the physical condition of nets and their perceived usefulness might contribute to a better understanding of net use patterns.

Provided sufficient LLINs are available in every household, usage targets would appear achievable considering that 78.8% of people with access to a LLIN in their household were found to make use of it. Regionally adapted strategies, potentially including complementary vector control interventions, may be required considering the discrepancies between survey locations, particularly the low usage despite high access in the Islands region. Supplementary net distribution strategies targeting pregnant women may help to improve LLIN use among this target group (currently 49.9%), which has remained below the 2011 programme target of 60%.

Insecticide-treated nets are known to convey a protective community effect beyond the individual user once a certain usage level in the general population has been achieved. Malaria models based on African data suggest that 35%-65% overall use can result in community-wide effects at least equivalent to that of personal protection [28]. Based on this threshold and at current levels of LLIN use (48.3%), a certain community-wide protective effect could be expected beyond the effect for the individual net user. This would particularly apply to the 35.1% of surveyed villages in which ≥60% LLIN use were reported. However, the mentioned models have not been parameterized with data from PNG, a transmission setting far more complex than most places in Africa.

### Methodological considerations

This survey applied a province-stratified sampling design, requiring weights to be applied during the analysis. While this approach is more cumbersome at the analysis stage, a probability proportional to size sampling was considered inadequate as reliable up-to-date population figures of census units were not available.

This publication used a complementary set of indicators (and their inverse as “coverage-gap”), including the “proportion of households with at least one LLIN per two people” and the “proportion of the population with access to a LLIN within their household”, the benefits of which have been discussed in detail by Kilian *et al.*[13]. While the latter indicator is less straightforward to calculate, both provide a more in-depth understanding of the LLIN coverage. In particular, the varying number of household members appears to render the Global Fund indicator “two or more LLIN per household” less useful than the assessment of households with at least one net per two household members. The access indicator, on the other hand, provides an excellent opportunity to estimate the proportion of people not using a LLIN despite having access to one. In the context of planning strategies for increasing net use, the distinction between deliberate non-use and lack of access to a net are crucial. In the PNG context, the population in the Islands region should be encouraged to use existing nets while in Momase and Highlands regions, additional nets are required to increase usage.

For the evaluation of the Global Fund programme, only country-level figures were requested [2]. In consideration of the diversity of PNG, a national figure is highly likely to mask sub-national heterogeneities. This was reflected, for example, in an analysis which found within-province disparities in child mortality [29]. A regional-level analysis of net coverage indicators was therefore performed, but the sample size did not allow for a higher resolution analysis. In order to assess equity of coverage (Figure 1), a PCA of household assets was performed – a method usually applied in settings where household income or expenditure data cannot be easily collected. Quintiles of PCA scores are generally presented to reflect household socio-economic status. Often, there is little discussion of the adequacy of this approach in a particular setting. This study has found the need for such dialogue through the occurrence of heterogeneities in contextual factors creating unexpected abnormalities in the results. This situation occurred after the construction of the wealth index, which resulted in a disproportionately high number of households in the Island region being assigned to the highest wealth quintile (Additional file 2: Figure S1). Particular household assets may be of very different value depending on the local geographical, environmental, or socio-cultural context. A typical example would be ownership of a boat or canoe, which would be essential for some (but not all) people living on the coast or on islands, as well as along large rivers; the value of a car, on the other hand, would be highly dependent on road access, which is independent of the geographical region. Similarly, ownership of certain livestock, such as pigs or goats, may be a sign of wealth but the value would depend heavily on cultural factors. Another abnormality was observed in the wealth index when stratified by certain assets, a phenomenon that can be considered as being due to “the richest man in the village”. This was a frequently observed phenomenon in the results where individuals were categorized as being in the highest indices of wealth in the country while still lacking numerous characteristics associated with wealth such as type of home, type of fuel used for cooking and toilet type. This is due to the unique difficulties in PNG wherein the assets and services most associated with wealth are still subject to availability. As such, individuals in remote places may not have the option for water being piped into their dwelling or a flushing toilet. Such contextual heterogeneities are difficult to identify and delineate in a large-scale survey and can consequently not easily be adjusted for in the analysis. Comparisons of outcomes between wealth quintiles should therefore be interpreted with caution.

## Conclusions

The repeated free LLIN distribution campaigns in PNG have led to substantial increases in all mosquito net indicators on a national level. Regional exceptions and shortfalls in specific target groups should be addressed in subsequent distribution rounds or with complementary interventions. Accessibility of villages no longer appears to be a major determinant of LLIN ownership, suggesting a better spatial coverage of the programme since 2009. Additional nets are required in areas where access is low, particularly the highly endemic Momase region, while the use of existing nets should be encouraged in the Islands region, where access is high but use remains low. Non-permanent household guests and people in high quality houses are currently poorly covered and may benefit from complementary vector control approaches, as may those people who continue to resist the regular use of a mosquito net.

## Competing interests

The authors declare that they have no competing interests.

## Authors’ contributions

MWH and IM developed the study, MWH and JP implemented the data collection, MWH and AAC analysed the data, YU was responsible for data management, and all co-authors contributed to data interpretation. MWH wrote the first draft of the manuscript and all co-authors contributed to and approved of the final version. All authors read and approved the final manuscript.

## Supplementary Material

Additional file 1 Variables used in the asset index and in the construction of a “high quality house” indicator. Click here for file

Additional file 2: Figure S1 Proportion of households by wealth quintile and region. Click here for file
