# Supplementary material for: Progress in mosquito net coverage in Papua New Guinea
Source: Malar J. 2014 Jun 24;13:242. doi: 10.1186/1475-2875-13-242 (PMC4077150; doi:10.1186/1475-2875-13-242)
Supplement: Additional file 1 — Variables used in the asset index and in the construction of a “high quality house” indicator. [file 1475-2875-13-242-S1.pdf]

Additional File 1: Household variables used for construction of wealth index

1. Main material of the floor
   - Earth/sand
   - Palm/bamboo/grass
   - Wood
   - Polished wood
   - Cement/tiles
2. Main material of the exterior walls
   - Bamboo / pitpit
   - Sago
   - Wood
   - Plywood
   - Masonite/Fibro
   - Cement or bricks
   - Iron sheets
3. Main material of the roof
   - Thatched grass
   - Sago palm leaves
   - Corrugated iron
   - Wood planks
   - Cement
4. Windows in main house
   - No windows
   - Windows, not screened
   - Windows, partly screened
   - Windows, all screened
5. Main source of drinking water
   - Surface water (river, pond, irrigation channel, etc.)
   - Open well (public/private)
   - Protected well (public/private)
   - Water tank
   - Piped into neighbourhood / public tap
   - Piped into dwelling
6. Main energy source for cooking
   - Firewood
   - Small twigs/tree branches/coconut shell
   - Kerosene
   - Gas
   - Electricity
7. Main source of lighting
   - None
   - Candle
   - Lantern
   - Pressure lamp/Coleman
   - Battery lantern
   - Solar power
   - Electricity

8. Ownership of household assets
    - Bed
    - Mattress
    - Table
    - Chair
    - Umbrella
    - Clothing cupboard
    - Coleman pressure lamp
    - Electric (battery) torch
    - Radio
    - Television
    - Mobile phone
    - Landline phone or wireless handset
    - Canoe, dugout (without motor)
    - Banana boat, ship (with motor)
    - Refrigerator
    - Bicycle / tricycle
    - Motorbike
    - Car or truck
9. Household ownership of livestock/animals (number of)
    - Chicken
    - Cassowaries
    - Goats and sheep
    - Pigs
    - Cows
    - Dogs
10. High quality house (see below)

Variables used for construction of "high quality house" indicator

1. Main source of drinking water
    - Piped into dwelling

AND

2. Type of toilet facility is available to the household
    - Own flushing toilet

AND

3. Main energy source for cooking
    - Gas or electricity
