# Supplementary material for: Progress in mosquito net coverage in Papua New Guinea
Source: Malar J. 2014 Jun 24;13:242. doi: 10.1186/1475-2875-13-242 (PMC4077150; doi:10.1186/1475-2875-13-242)
Supplement: Additional file 2: Figure S1 — Proportion of households by wealth quintile and region. [file 1475-2875-13-242-S2.pdf]

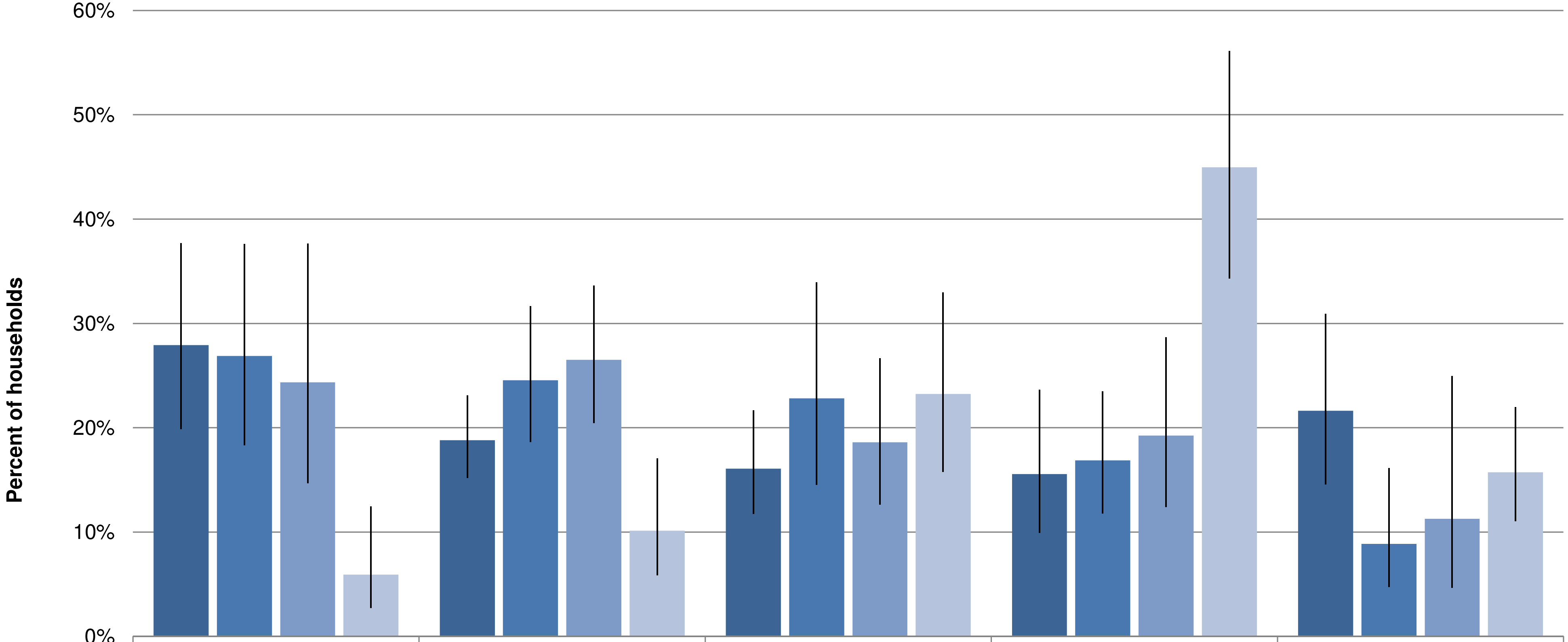

	Lowest	Second	Third	Fourth	Highest
Southern	27.91%	18.81%	16.09%	15.56%	21.63%
Highlands	26.87%	24.55%	22.82%	16.88%	8.88%
Momase	24.35%	26.50%	18.61%	19.25%	11.28%
Island	5.93%	10.14%	23.24%	44.97%	15.72%

Wealth quintile

Additional File 2: Proportion of households by wealth quintile and region
Error bars represent 95% confidence intervals.
